# Leisure-time and commuting physical activity and changes in psychological distress: a prospective study among young and early midlife Finnish employees

**DOI:** 10.1177/14034948251391982

**Published:** 2025-11-12

**Authors:** Lari Järvensivu, Tea Lallukka, Ville Päivärinne, Ossi Rahkonen

**Affiliations:** 1 Department of Public Health, University of Helsinki, Helsinki, Finland; 2 Finnish Institute for Health and Welfare, Helsinki, Finland

**Keywords:** Leisure-time physical activity, changes in psychological distress, young and early midlife adults, prospective cohort study, occupational cohort, moderate to vigorous physical activity, mental health

## Abstract

**Aims:**

Physical activity is linked to better mental health. However, the associations of physical activity and subsequent changes in mental health are poorly understood. We aimed to investigate associations between leisure-time and commuting physical activity and changes in mental health as indicated by psychological distress using the Finnish Helsinki Health Study data collected in 2017 (phase 1) and 2022 (phase 2) among young and early midlife employees (*n*=3135).

**Methods:**

Leisure-time and commuting physical activity was measured with questions pertaining to four distinct levels of intensity and formed a four-class variable. We measured psychological distress using the emotional wellbeing subscale from the RAND-36 questionnaire, with scores ranging from 0 to 100 (higher scores indicate better mental health). We used a cut point of 52 to compute a four-class variable of change in psychological distress. We used multinomial regression models while adjusting for key covariates to estimate average marginal effects and odds ratios.

**Results:**

At phase 1 the prevalence of lowest activity was 15.6% and the prevalence of highest activity was 21.3%. The prevalence of repeated psychological distress was 5.9% and 75.6% for those repeatedly without psychological distress. After adjustments, low activity was associated with a 12 percentage point lower predicted probability of being repeatedly without psychological distress (average marginal effect −0.12, 95% confidence interval −0.18 to −0.06) when comparing with very high activity. The same association was weaker for medium activity.

**Conclusions:**

**The results showed that lower activity was associated with psychological distress which indicates that higher leisure-time and commuting physical activity levels may help to maintain mental health**.

## Background

Physical activity is seen as a major contributor to health. The current physical activity guidelines recommend meeting or exceeding 150 minutes of moderate (3–5.9 metabolic equivalents (METs)) or 75 minutes of vigorous (⩾6 METs) intensity physical activity per week or an equivalent combination of both [1]. However, the prevalence of not meeting these guidelines has risen from 26.4% in 2010 to 31.3% in 2022 [2]. This rising prevalence of insufficient physical activity poses a major public health challenge to both physical and mental wellbeing.

Mental health is also a major global concern. According to the World Health Organization (WHO), in 2019, 13% of the world’s population was suffering from a mental disorder. Anxiety and depression were the primary contributors, accounting for 60% of the total burden [3]. Psychological distress, closely related to depression and anxiety is defined as mental and physical symptoms that are associated with normal fluctuations in mood which in some cases may be indicative of an underlying mood or anxiety disorder [4]. It is often measured with instruments such as the MHI-5, which can identify individuals meeting diagnostic criteria for these disorders [5]. Additionally, psychological distress predicts long-term sickness absence and work disability [6,7].

Among those aged 20–34 years in the Finnish working population the prevalence of psychological distress, as measured by the MHI-5, was 15.6% for women and 12.0% for men [8]. Simultaneously, Finnish estimates from 2022 reveal that among 20 to 39-year-olds, 48.2% of men and 57.8% of women were unable to meet current guidelines for both the aerobic and muscle-strengthening component [9]. Evidence from an umbrella review of randomised controlled trials have proved physical activity to be effective for depression, anxiety and psychological distress [10]. Similarly, a previous umbrella review of prospective cohort studies found it to have a protective effect against depression and anxiety disorders [11]. However, there are clearly less previous prospective cohort studies specifically examining psychological distress.

Two studies using the MHI-5 linked increases in leisure-time physical activity (LTPA) with decreases in psychological distress—the Dutch study found this only in men, while the French study found it in both genders [12,13]. However, these studies did not provide information on how different levels of LTPA relate to changes in psychological distress. Three other prospective studies have used different measures of psychological distress. An Australian study found that higher moderate to vigorous physical activity (MVPA) frequency in any domain was associated with lower distress [14]. A Norwegian study reported similar findings for hours spent in light and moderate intensity LTPA [15]. A British study found that higher intensity, but not frequency or total LTPA, predicted lower psychological distress [16]. However, a limitation of these studies was that they did not use a measure, such as MET-hours, that captures equivalent activity levels across different patterns of intensity, frequency, and duration.

### Aims

The present study aimed to investigate associations between different levels of leisure-time and commuting physical activity (LCPA) and changes in psychological distress in the Helsinki Health Study (HHS) young and early midlife employees’ cohort. Adding to the current literature, we considered the total amount of LCPA (in MET-hours) and the intensity of LCPA simultaneously and examined how they relate to changes in psychological distress, while adjusting for covariates based on previous studies [17,18].

## Methods

The HHS is an ongoing research project on the employees of the City of Helsinki, Finland. The baseline dataset for this study comes from a survey conducted on 19–39-year-old employees in 2017 (phase 1) [19]. Besides basic demographic questions the survey included questions on participants’ LCPA, psychological distress and other health-related factors, work, and social life. This survey was sent to all those who had been employed for at least 4 months and were working halftime or more for the City of Helsinki (*n*=11,459). The respondents could respond either online via a secure server or use a mailed survey [19]. For the non-respondents a shorter telephone interview was conducted, which resulted in a final response rate of 51.5% (*n*=5898, 78.5% women) [19]. However, the telephone respondents (*n*=787) were excluded because the questionnaire used for telephone interviews did not include questions on LCPA.

The follow-up study conducted in 2022 (phase 2) targeted the phase 1 survey respondents. The phase 2 survey had a response rate of 60% (*n*=3520). After excluding non-respondents to phase 2, there was no missing information regarding age or gender. Finally, we only included participants without missing data on baseline LCPA (2017) and psychological distress (2017 and 2022). This process, described in detail in Figure 1, comprised our final analytical sample of 3135 participants (80.2% women).

**Figure 1.**
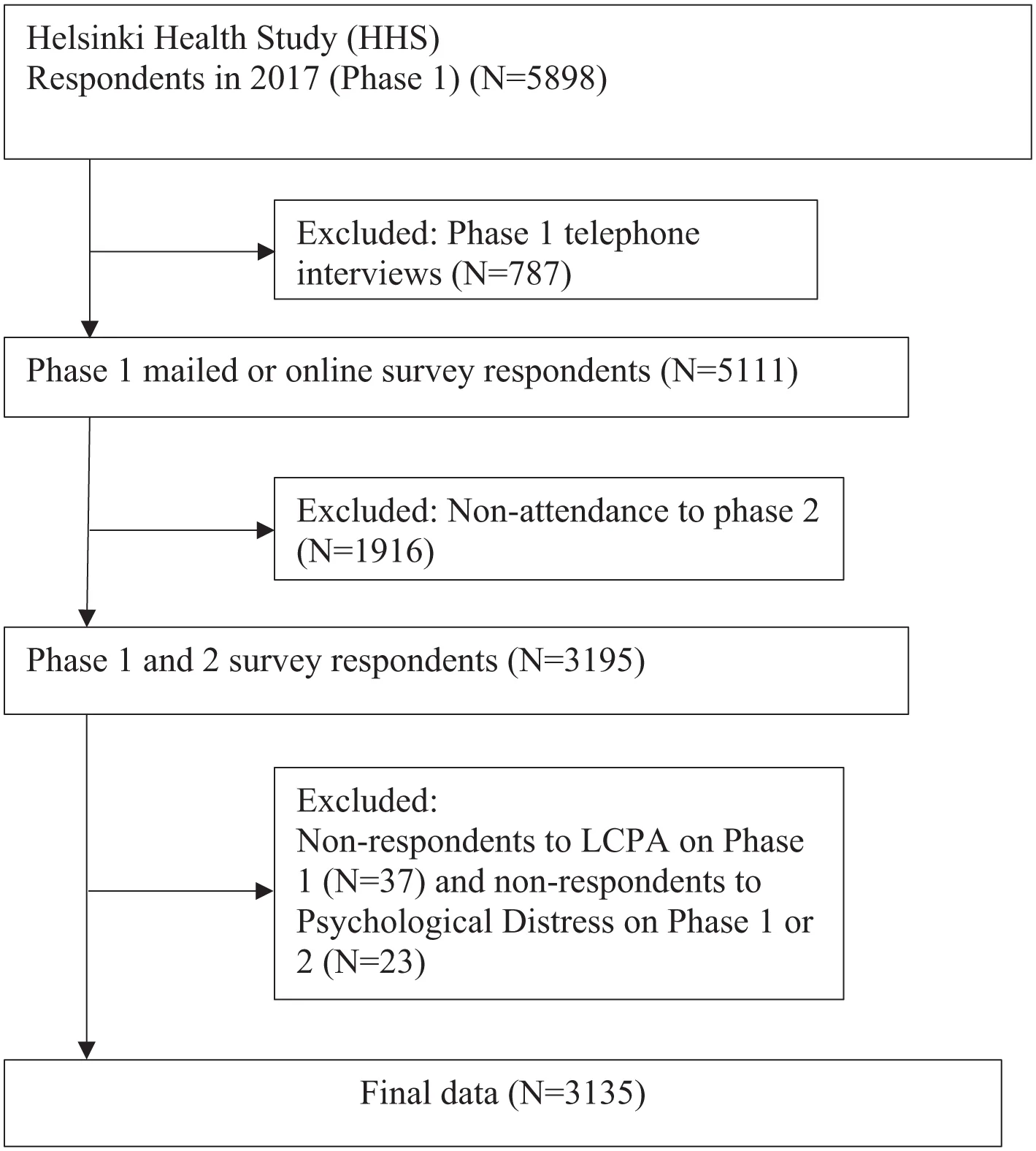
Flow chart of the exclusions leading to the final sample of participants. LCPA: Leisure-time and commuting physical activity.

### Leisure-time and commuting physical activity

Participants’ baseline (phase 1) weekly LTPA was measured with four questions pertaining to four levels of intensity equivalent to walking, brisk walking, jogging, and running. The same four questions were also used to measure commuting activity. These were combined because after analysing these as separate variables we saw that commuting did not show any associations with psychological distress, and the associations for LTPA were slightly weaker than for combined LCPA (Supplemental Figure 3). We calculated an approximation of each participant’s weekly physical activity using MET-hours, a measure which combines the duration and intensity of exercise. We used the following grouping for LCPA: (a) low activity (under 20 MET-hours per week equivalent to 5 hours of walking); (b) medium activity (20 MET-hours or more per week); (c) high activity (20–80 MET-hours per week including vigorous activity); (d) very high activity (over 80 MET-hours per week including vigorous activity equivalent to 10 hours of walking and 4 hours jogging) in line with our previous studies [17].

### Psychological distress

To measure psychological distress, we used the emotional wellbeing subscale, from the RAND-36 questionnaire [20]. The emotional wellbeing scale is identical in content to the more widely used MHI-5, although there are minor differences between the scales in the exact steps of computation the resulting scores are identical (see Supplemental Equation 1.). This justified us referring to our outcome as psychological distress, which has been done in previous studies using the MHI-5 [8]. The MHI-5 has also been validated in a sample representative of the Finnish general population, and national estimates by the Finnish Institute of Health and Welfare (THL) also rely on the MHI-5 [21]. The scale consists of five questions pertaining to psychological distress. These can be viewed in our supplemental file (Supplemental Table III). According to RAND scoring guidelines answers from the singular questions were transformed and averaged into a final score of psychological distress that ranged from 0 to 100 [20]. We used 52 as our cut-off based on previous research from THL [8,21], dichotomising the participants into either having psychological distress (⩽52) or not having psychological distress (>52) at both phase 1 and phase 2. This resulted in four change categories: (a) not having psychological distress in 2017 and 2022; (b) not having psychological distress in 2017 and having psychological distress in 2022; (c) having psychological distress in 2017 and not having psychological distress in 2022; and (d) having psychological distress in 2017 and 2022.

### Covariates

The covariates were chosen based on previous studies and previously found associations with either psychological or LTPA [17,18]. We included information on participants’ gender, age, education, employment status, marital status, physical and mental strenuousness of work, alcohol and tobacco consumption, fresh vegetable consumption, body mass index (BMI) and sedentary time all derived from the baseline survey (phase 1). Participants’ education level was used as an indicator for their socioeconomic position. Education level comprised a three-category variable: ‘low’ (upper secondary school or lower), ‘intermediate’ (bachelor’s degree) and ‘high’ (master’s degree or higher). Employment status resulted in a two-category variable: ‘working’ (full or part-time) and ‘not working’ (e.g. parental leave or other). Marital status was dichotomised into ‘married or cohabiting’ and ‘not married or cohabiting’ based on five response alternatives. Physical and mental strenuousness of work were both dichotomised into a high (‘rather strenuous’ and ‘very strenuous’) and a low (‘very light’ and ‘rather light’) group each based on four response alternatives. Alcohol consumption was divided into ‘binge drinkers’ and ‘not binge drinkers’. Binge drinking was defined as consuming more than six portions of alcohol on one occasion at least once a week for men and once a month for women. Tobacco consumption was divided into ‘daily or occasional’ smokers and ‘non-smokers or ex-smokers’. Fresh vegetable consumption was dichotomised in to ‘daily’ and ‘not daily’. BMI was calculated from participants’ self-reported height and weight. For a concise description of participants’ BMI distribution three groups were formed: BMI under 25 (with healthy weight and underweight), BMI between 25 and 30 (with overweight) and BMI over 30 (with obesity). Only 1.7% of the sample were underweight (BMI <18.5) and for this reason they were combined into the same category with those with healthy weight. In the regression models BMI was used as a continuous covariate and the missing values (*n*=27) were replaced by the median BMI of the participants. Sedentary time was dichotomised into ‘low or intermediate’ and ‘high (>8.5 h)’. Any missing data on the covariates was replaced by what we predicted would be the lowest risk category, following previous procedures [22].

### Statistical analyses

Counts and percentages of baseline (phase 1) descriptive characteristics are shown separately for men and women in Table I. We ran a chi-squared tests for crosstabulations of psychological distress change groups and phase 1 LCPA groups and the covariates (Table II). For the main analysis we ran a multinomial regression to investigate the associations between baseline LCPA groups and psychological distress change groups using average marginal effects (AMEs) and odds ratios (ORs).

**Table I.** Descriptive characteristics of the Helsinki Health Study participants in 2017 (phase 1).

	All, *n* (%)	Women, *n* (%)	Men, *n* (%)	*P* value^a^
	3135 (100.0)	2515 (80.2)	620 (19.8)	0.005
Age, years				
19–29	893 (28.5)	737 (29.3)	156 (25.2)	0.005
30–34	1058 (33.7)	863 (34.3)	195 (31.5)	
35–40	1184 (37.8)	915 (36.4)	269 (43.4)	
Marital status				
Married or cohabiting	2078 (66.3)	1635 (65.0)	443 (71.5)	0.003
Not in a relationship	1057 (33.7)	880 (35.0)	177 (28.5)	
Education				
High	981 (31.3)	792 (31.5)	189 (30.5)	<0.001
Intermediate	1195 (38.1)	1014 (40.3)	181 (29.2)	
Low	959 (30.6)	709 (28.2)	250 (40.3)	
Employment status				
Working	2796 (89.2)	2199 (87.4)	597 (96.3)	<0.001
Not working	339 (10.8)	316 (12.6)	23 (3.7)	
Mental strenuousness of work				
Low	709 (22.6)	516 (20.5)	193 (31.1)	<0.001
High	2426 (77.4)	1999 (79.5)	427 (68.9)	
Physical strenuousness of work				
Low	2145 (68.4)	1670 (66.4)	475 (76.6)	<0.001
High	990 (31.6)	845 (33.6)	145 (23.4)	
Alcohol consumption				
Not a binge drinker	2588 (82.6)	2062 (82.0)	526 (84.8)	0.106
Binge drinker	547 (17.4)	453 (18.0)	94 (15.2)	
Tobacco consumption				
Non-smokers or ex-smokers	2426 (77.4)	1952 (77.6)	474 (76.5)	0.571
Daily or occasional	709 (22.6)	563 (22.4)	146 (23.5)	
Fresh vegetable consumption				
Daily	2194 (70.0)	1855 (73.8)	339 (54.7)	<0.001
Not daily	941 (30.0)	660 (26.2)	281 (45.3)	
Body mass index				
<25 with healthy weight	1835 (58.5)	1554 (61.8)	281 (45.3)	<0.001
25–30 with overweight	834 (26.6)	589 (23.4)	245 (39.5)	
>30 with obesity	466 (14.9)	372 (14.8)	94 (15.2)	
Sedentary time				
Low or intermediate	221 (7.0)	182 (7.2)	39 (6.3)	0.461
High (>8.5 h)	2914 (93.0)	2333 (92.8)	581 (93.7)	
LCPA				
Very high activity	669 (21.3)	489 (19.4)	180 (29.0)	<0.001
High activity	1202 (38.3)	962 (38.3)	240 (38.7)	
Medium activity	775 (24.7)	659 (26.2)	116 (18.7)	
Low activity	489 (15.6)	405 (16.1)	84 (13.5)	
Phase 1: Psychological distress				
No	2669 (85.1)	2144 (85.2)	525 (84.7)	0.768
Yes	466 (14.9)	371 (14.8)	95 (15.3)	
Phase 2: Psychological distress				
No	2647 (84.4)	2139 (85.0)	508 (81.9)	0.064
Yes	488 (15.6)	376 (15.0)	112 (18.1)	

a The *P* values indicate the significance of a chi-square test assessing the probability of observing the given distributions under the assumption that there is no difference in prevalence between women and men across the variable categories.

LCPA: leisure-time and commuting physical activity.

**Table II.** The distributions of psychological distress change categories across LCPA and covariate groups among the Helsinki Health Study participants in 2017 (phase 1).

	Change in psychological distress				
	No→No^a^	No→Yes^a^	Yes→No^a^	Yes→Yes^a^	
	*n* (%)	*n* (%)	*n* (%)	*n* (%)	*P* value^b^
Total	2369 (75.6)	278 (8.9)	300 (9.6)	188 (5.9)	
Age, years					
19–29	643 (27.1)	106 (38.1)	87 (29.0)	57 (30.3)	0.013
30–34	817 (34.5)	82 (29.5)	93 (31.0)	66 (35.1)	
35–40	909 (38.4)	90 (32.4)	120 (40.0)	65 (34.6)	
Marital status					
Married or cohabiting	1643 (69.4)	166 (59.7)	188 (62.7)	81 (43.1)	<0.001
Not in a relationship	726 (30.6)	112 (40.3)	112 (37.3)	107 (56.9)	
Education					
High	778 (32.8)	76 (27.3)	83 (27.7)	44 (23.4)	0.001
Intermediate	677 (28.6)	96 (34.5)	110 (36.7)	76 (40.4)	
Low	914 (38.6)	106 (38.1)	107 (35.7)	68 (36.2)	
Employment status					
Working	2104 (88.8)	254 (91.4)	264 (88.0)	174 (92.6)	0.225
Not working	265 (11.2)	24 (8.6)	36 (12.0)	14 (7.4)	
Mental strenuousness of work					
Low	577 (24.4)	41 (14.7)	64 (21.3)	27 (14.4)	<0.001
High	1792 (75.6)	237 (85.3)	236 (78.7)	161 (85.6)	
Physical strenuousness of work					
Low	1637 (69.1)	193 (69.4)	196 (65.3)	119 (63.3)	0.236
High	732 (30.9)	85 (30.6)	104 (34.7)	69 (36.7)	
Alcohol consumption					
Not a binge drinker	1990 (84.0)	213 (76.6)	251 (83.7)	134 (71.3)	<0.001
Binge drinker	379 (16.0)	65 (23.4)	49 (16.3)	54 (28.7)	
Tobacco consumption					
Non or ex-smokers	1883 (79.5)	202 (72.7)	217 (72.3)	124 (66.0)	<0.001
Daily or occasional	486 (20.5)	76 (27.3)	83 (27.7)	64 (34.0)	
Fresh vegetables					
Daily	1710 (72.2)	172 (61.9)	210 (70.0)	102 (54.3)	<0.001
Not daily	659 (27.8)	106 (38.1)	90 (30.0)	86 (45.7)	
Sedentary time					
Low or intermediate	148 (6.2)	28 (10.1)	27 (9.0)	18 (9.6)	0.026
High (>8.5 h)	2221 (93.8)	250 (89.9)	273 (91.0)	170 (90.4)	
Body mass index					
<25 with healthy weight	1432 (60.4)	149 (53.6)	161 (53.7)	93 (49.5)	<0.001
25–30 with overweight	630 (26.6)	73 (26.3)	75 (25.0)	56 (29.8)	
>30 with obesity	307 (13.0)	56 (20.1)	64 (21.3)	39 (20.7)	
LCPA					
Very high activity	535 (22.6)	44 (15.8)	58 (19.3)	32 (17.0)	<0.001
High activity	956 (40.4)	95 (34.2)	98 (32.7)	53 (28.2)	
Medium activity	556 (23.5)	78 (28.1)	88 (29.3)	53 (28.2)	
Low activity	322 (13.6)	61 (21.9)	56 (18.7)	50 (26.6)	

a No→No: Not having psychological distress in 2017 and 2022.

No→Yes: Not having psychological distress in 2017 but having it in 2022.

Yes→No: Having psychological distress in 2017 but not having it in 2022.

Yes→Yes: Having psychological distress in 2017 and 2022.

b The *P* values indicate the significance of a chi-square test assessing the probability of observing the given distributions under the assumption that there is no difference in prevalence between categories of psychological distress change across the variable categories.

LCPA: leisure-time and commuting physical activity.

We used two multinomial regression models for our main analysis. The first, model 1 was adjusted for age and gender. The second, model 2 was adjusted further for marital status, education, employment status, mental and physical strenuousness of work, BMI, sedentary time, fresh vegetable consumption, tobacco consumption and alcohol consumption. AMEs are based on the predicted probabilities given by the multinomial regression model. They tell the difference between two categories of the predictor variable in percentage points of predicted probability. We used pooled data as the male sample was small and the interaction between gender and LCPA class was not statistically significant (*P*=0.106). Separate associations for men and women can be found in our supplemental file (Supplemental Figure 1). All analyses were conducted using R version 4.4.0.

## Results

The descriptive characteristics of study participant are shown in Table I. Men were more likely to belong to the very high activity group (19.4% vs. 29.0%), whereas women were more likely to belong to the medium activity group (26.2% vs. 18.7%). There were no clear differences between men and women in the prevalences of psychological distress in either phase 1 or 2. However, there were differences between men and women regarding most covariates as shown in Table II. The prevalences of high and very high activity were highest among those without psychological distress at both phases, while the prevalence of low activity was highest among those with psychological distress at both phases. Additionally, clear differences in the prevalence of psychological distress change groups were observed across nearly all covariates (Table II).

Table III presents the AMEs of different levels of LCPA on the probability of belonging to each of the four psychological distress change groups, using the very high group as the reference. In model 1, medium activity was associated with an approximately 9 percentage point lower predicted probability of repeatedly not reporting psychological distress (AME = −0.9, 95% confidence interval (CI) –0.13 to −0.05). Low activity was similarly associated with lower predicted probability (AME = −0.15, 95% CI −0.20 to −0.09) of repeatedly not reporting psychological distress. Additionally, low activity was associated with an increased probability of repeated psychological distress (AME = 0.06, 95% CI 0.03 to 0.09). The probability of not reporting psychological distress in 2017 but reporting it in 2022 was also higher in the low activity group and medium activity group (AME = 0.06, 95% CI 0.03 to 0.10 for low; AME = 0.04, 95% CI 0.01 to 0.7 for medium). The associations remained after adjustment for covariates as depicted in Table III (model 2). These associations are also depicted using ORs in Figure 2.

**Table III.** Average marginal effects (AMEs) of leisure-time and commuting physical activity categories on psychological distress change.

	Psychological distress change group			
	No→No^a^	No→Yes^a^	Yes→No^a^	Yes→Yes^a^
	AME (95% CI)	AME (95% CI)	AME (95% CI)	AME (95% CI)
Model 1^b^				
Very high activity	Ref.	Ref.	Ref.	Ref.
High activity	−0.01 (–0.05–0.03)	0.01 (–0.01–0.04)	0 (–0.03–0.02)	0 (–0.02–0.02)
Medium activity	−0.09 (–0.13– –0.05)	0.04 (0.01–0.07)	0.03 (0–0.06)	0.02 (0–0.05)
Low activity	−0.15 (–0.2– –0.1)	0.06 (0.03–0.1)	0.03 (–0.01–0.06)	0.06 (0.03–0.09)
Model 2^b^				
Very high activity	Ref.	Ref.	Ref.	Ref.
High activity	−0.01 (–0.05–0.03)	0.01 (–0.01–0.04)	0 (–0.03–0.03)	0 (–0.02–0.02)
Medium activity	−0.07 (–0.12– –0.03)	0.03 (0–0.06)	0.02 (–0.01–0.06)	0.02 (–0.01–0.04)
Low activity	−0.12 (–0.17– –0.07)	0.05 (0.02–0.09)	0.03 (–0.01–0.06)	0.04 (0.01–0.07)

a No→No: Not having psychological distress in 2017 and 2022.

No→Yes: Not having psychological distress in 2017 but having it in 2022.

Yes→No: Having psychological distress in 2017 but not having it in 2022.

Yes→Yes: Having psychological distress in 2017 and 2022.

b Model 1 was adjusted for age and gender and model 2 further for marital status, education, employment status, mental and physical strenuousness of work, body mass index, sedentary time, fresh vegetable consumption, tobacco consumption and alcohol consumption.

CI: confidence interval.

**Figure 2.**
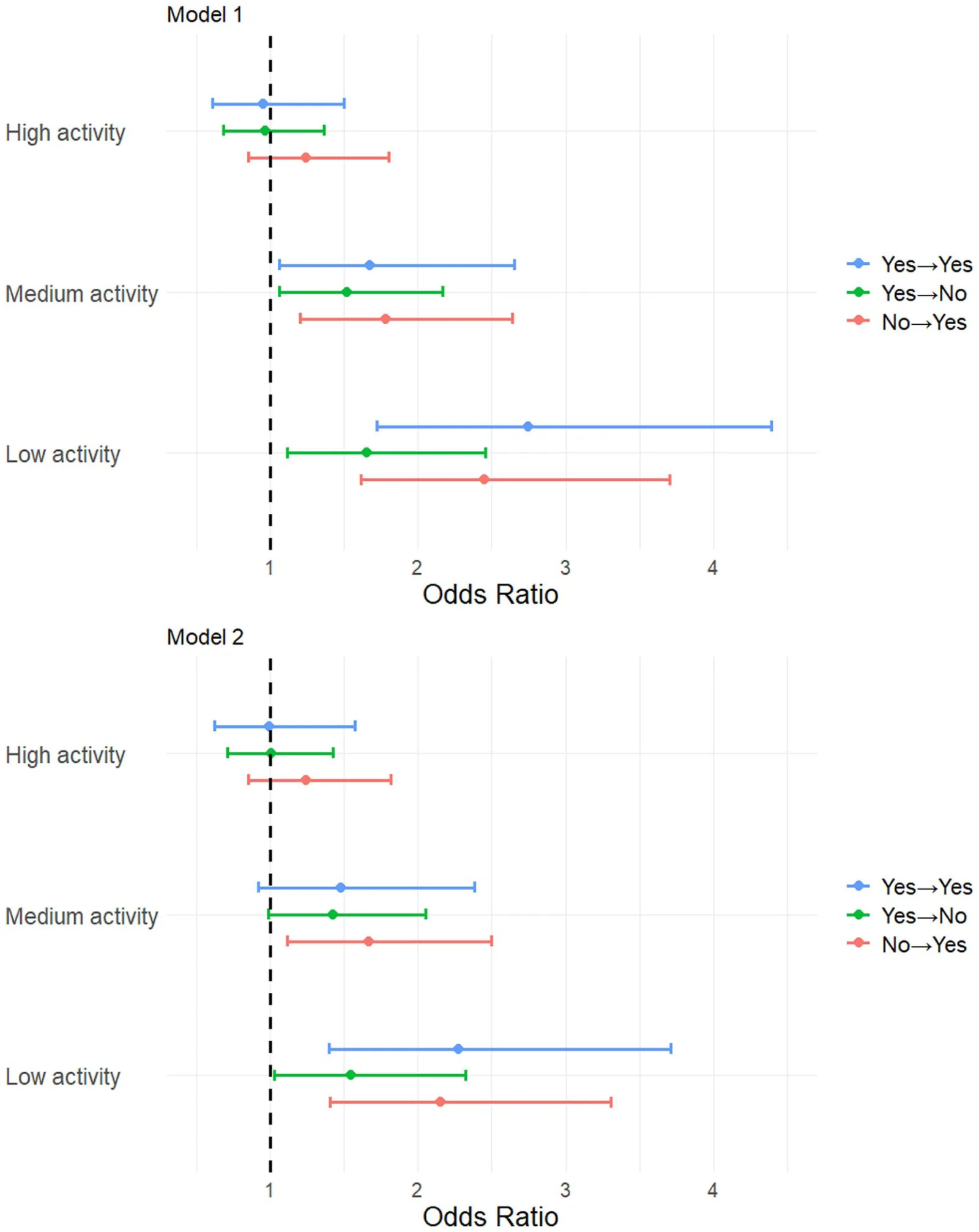
Odds ratios of psychological distress change across categories of leisure-time and commuting physical activity. No→No: Not having psychological distress in 2017 and 2022. No→Yes: Not having psychological distress in 2017 but having it in 2022. Yes→No: Having psychological distress in 2017 but not having it in 2022. Yes→Yes: Having psychological distress in 2017 and 2022. Model 1 was adjusted for age and gender and model 2 further for marital status, education, employment status, mental and physical strenuousness of work, body mass index (BMI), sedentary time, fresh vegetable consumption, tobacco consumption and alcohol consumption. Reference categories are very high activity for LCPA and No→No for psychological distress change.

## Discussion

We aimed to study associations between LCPA levels and changes in psychological distress in young and early midlife employees using two time points: a baseline (phase 1) and a 5-year follow-up (phase 2). Our results revealed that both low and medium activity were associated with a lower predicted probability of repeatedly not having psychological distress. Similarly, we also found an association between low and medium activity and changing from not having psychological distress at phase 1 to having it at phase 2. Additionally, belonging to the low activity group was associated with a higher predicted probability of having repeated psychological distress.

The inclusion of vigorous physical activity seemed to explain our results for two reasons. First, the low, medium, and high activity groups averaged 11.1 (SD 5.5), 48.3 (SD 35.6) and 50.9 (SD 15.9) MET-hours per week, respectively, showing a much larger difference between the low and medium activity groups compared with the difference between the medium and high activity groups. This suggests that the difference did not emerge only due to the differences in total MET-hours but rather due to including vigorous physical activity. Additionally, a further analysis examining all intensities of LCPA as separate variables showed the strongest association between vigorous physical activity and psychological distress (Supplemental Figure 2).

The two previous studies using the identical measure MHI-5 found a tentative role for LTPA in managing psychological distress. A French study found that increases in total LTPA were associated with less psychological distress [12], while a Dutch study found the same association, but only among men [13]. As these two studies examined changes in LTPA we conducted an additional analysis adjusting for LCPA in 2022. After adjusting for LCPA in 2022 only two associations remained—low activity was associated with a higher predicted probability of repeatedly having psychological distress and a lower predicted probability of repeatedly not having psychological distress (Supplemental Table I). Three other prospective studies using different measures of psychological distress found physical activity to be associated with lower psychological distress [14–16]. The findings from these earlier studies and the present study are consistent with those of a previous umbrella review of prospective cohort studies, which concluded that physical activity has a protective effect against depression and anxiety disorders [11].

There are many alleged mechanisms explaining the benefits of physical activity on mental health. Psychosocial mechanisms explaining the effects for depression include the promotion of self-esteem, social support and self-efficacy [23]. However, different mechanisms may operate depending on the intensity of physical activity. For example, high-intensity physical activity may increase neuroplasticity more than lighter intensities [23]. However, other mechanisms such as reduced inflammation might be more prominent with lighter intensities [23]. The present study, and previous studies on psychological distress with any examination of physical activity intensity, suggest that higher intensities are more effective in young and early midlife adults but also in ageing adults [15,16].

There were some limitations in our study. First, there are some characteristics of the study sample that influence the generalisability of the results. Our sample consisted of public sector employees and thus were in the majority women (80.5%). Although this reflects the gender distribution of the study population [19] this meant that women dominated the associations. Attrition to phase 2 was higher among those with lower education and younger age which might have biased the results. However, there were no differences regarding LCPA, psychological distress or gender between respondents and non-respondents to phase 2 (Supplemental Table II). Additionally, a substantial minority (*n*=339) of our sample was not currently working at the time of the phase 1 survey. The most common reasons for not currently working were parental leave and studying (*n*=307). However, there were virtually no differences in the results when those not currently working were excluded (data not shown). Second, we used self-report to measure participants’ LCPA, which is vulnerable to overreporting bias and socially desirable answers, although using objective measures in such a large sample is not feasible. Additionally, we used a non-validated measure of physical activity. However, previous research has shown that no physical activity questionnaire is clearly superior [24].

The present study also had important strengths. First, our prospective study design attenuated the possible effect of reverse causality and allowed the examination of the associations between LCPA and changes in psychological distress. Second, we used a widely recognised and validated measurement of psychological distress, the RAND-36 emotional wellbeing scale [12,13,17,20], and the identical MHI-5 has been deemed reliable at predicting a range of mental health outcomes [5–7]. Third, we were able to control for many important covariates following previous procedures and previously found associations with LCPA or psychological distress [8,17,18]. However, we cannot rule out the possibility of residual confounding from unmeasured or unknown factors. Finally, our LCPA measure allowed us to capture light, moderate and vigorous intensities whereas most previous studies have examined only MVPA [12–14].

## Conclusions

The present study further strengthens the existing evidence by examining associations between LCPA and changes in psychological distress while controlling for important sociodemographic and health-related behavioural factors. The results suggest that higher levels of physical activity and the inclusion of vigorous intensity, are important for managing psychological distress. Future studies could further examine how different intensities of LCPA are associated with psychological distress. The prevalence of psychological distress and mental disorders in the general and working population are notable impingements on the flourishing of society and individuals. Consequently, supporting policies and interventions aimed at increasing LCPA could enhance the populations’ mental health while providing physical health benefits.

## Supplemental Material

sj-docx-1-sjp-10.1177_14034948251391982 – Supplemental material for Leisure-time and commuting physical activity and changes in psychological distress: a prospective study among young and early midlife Finnish employees Supplemental material, sj-docx-1-sjp-10.1177_14034948251391982 for Leisure-time and commuting physical activity and changes in psychological distress: a prospective study among young and early midlife Finnish employees by Lari Järvensivu, Tea Lallukka, Ville Päivärinne and Ossi Rahkonen in Scandinavian Journal of Public Health

sj-tiff-2-sjp-10.1177_14034948251391982 – Supplemental material for Leisure-time and commuting physical activity and changes in psychological distress: a prospective study among young and early midlife Finnish employees Supplemental material, sj-tiff-2-sjp-10.1177_14034948251391982 for Leisure-time and commuting physical activity and changes in psychological distress: a prospective study among young and early midlife Finnish employees by Lari Järvensivu, Tea Lallukka, Ville Päivärinne and Ossi Rahkonen in Scandinavian Journal of Public Health

sj-tiff-3-sjp-10.1177_14034948251391982 – Supplemental material for Leisure-time and commuting physical activity and changes in psychological distress: a prospective study among young and early midlife Finnish employees Supplemental material, sj-tiff-3-sjp-10.1177_14034948251391982 for Leisure-time and commuting physical activity and changes in psychological distress: a prospective study among young and early midlife Finnish employees by Lari Järvensivu, Tea Lallukka, Ville Päivärinne and Ossi Rahkonen in Scandinavian Journal of Public Health

sj-tiff-4-sjp-10.1177_14034948251391982 – Supplemental material for Leisure-time and commuting physical activity and changes in psychological distress: a prospective study among young and early midlife Finnish employees Supplemental material, sj-tiff-4-sjp-10.1177_14034948251391982 for Leisure-time and commuting physical activity and changes in psychological distress: a prospective study among young and early midlife Finnish employees by Lari Järvensivu, Tea Lallukka, Ville Päivärinne and Ossi Rahkonen in Scandinavian Journal of Public Health

## Data Availability

Data can be obtained through reasonable requests. The Helsinki Health Study survey data cannot be publicly released due to stringent data protection laws and regulations. Use of the data is restricted to scientific research and collaboration with the research group’s partners, subject to a reasonable request and study plan. Further details regarding the availability of the survey data can be obtained by contacting the Helsinki Health Study research group at kttl-hhs@helsinki.fi.
