# Supplementary material for: Leisure-time and commuting physical activity and changes in psychological distress: a prospective study among young and early midlife Finnish employees
Source: Scand J Public Health. 2025 Nov 12;54(8):873–82. doi: 10.1177/14034948251391982 (PMC13710729; doi:10.1177/14034948251391982)
Supplement: Supplementary file 1 [file sj-docx-1-sjp-10.1177_14034948251391982.docx]

**Table S1.** Average Marginal Effects (AME) of leisure-time and commuting physical activity (LCPA) categories on psychological distress change in model 1 with additional adjustment for LCPA in 2022 (n = 2922)

|  | **Psychological distress change group** |  |  |  |
| --- | --- | --- | --- | --- |
|  | **No->No^a^** | **No->Yes^a^** | **Yes->No^a^** | **Yes ->Yes^a^** |
|  | **AME (95% CI)** | **AME (95% CI)** | **AME (95% CI)** | **AME (95% CI)** |
| **Model** **1^b^** |  |  |  |  |
| Very high activity | Ref. | Ref. | Ref. | Ref. |
| High activity | -0.01 (-0.05 – 0.03) | 0.02 (-0.01 – 0.05) | -0.01 (-0.04 – 0.02) | 0 (-0.02 – 0.02) |
| Medium activity | -0.03 (-0.11 – 0.04) | 0.01 (-0.04 – 0.05) | 0 (-0.06 – 0.05) | 0.03 (-0.02 – 0.07) |
| Low activity | -0.08 (-0.15 – 0.00) | 0.03 (-0.02 – 0.08) | 0 (-0.05 – 0.05) | 0.05 (0 .00– 0.1) |

***a:*** *No->No: Not having psychological distress in 2017 and 2022*

*No->Yes: Not having psychological distress in 2017 but having it in 2022*

*Yes->No: Having psychological distress in 2017 but not having it in 2022*

*Yes ->Yes: Having psychological distress in 2017 and 2022*

***b:*** *Model 1 was adjusted for age and gender*

**Table S2.** Participant attrition between Phase 1 and Phase 2.

|  |  | Response to Phase 2 survey | |  |
| --- | --- | --- | --- | --- |
|  | Overall (n(%) | No (n(%) | Yes (n(%) | *p^a^* |
|  | 5898 (100) | 2378 (40.3) | 3520 (59.7) |  |
| Gender |  |  |  |  |
| Woman | 4630 (78.5) | 1839 (77.3) | 2791 (79.3) | 0.078 |
| Man | 1268 (21.5) | 539 (22.7) | 729 (20.7) |  |
| Age |  |  |  |  |
| 19-29 | 1868 (31.7) | 875 (36.9) | 993 (28.2) | <0.001 |
| 30-34 | 2001 (33.9) | 809 (34.1) | 1192 (33.9) |  |
| 35-40 | 2025 (34.4) | 690 (29.1) | 1335 (37.9) |  |
| Education |  |  |  |  |
| Low | 2106 (35.8) | 1006 (42.4) | 1100 (31.3) | <0.001 |
| Intermediate | 2089 (35.5) | 771 (32.5) | 1318 (37.5) |  |
| High | 1688 (28.7) | 595 (25.1) | 1093 (31.1) |  |
| LCPA^b^ |  |  |  |  |
| Low activity | 781 (15.5) | 284 (15.1) | 497 (15.7) | 0.148 |
| Medium activity | 1260 (25.0) | 481 (25.6) | 779 (24.7) |  |
| High activity | 1889 (37.5) | 676 (35.9) | 1213 (38.4) |  |
| Very high activity | 1110 (22.0) | 441 (23.4) | 669 (21.2) |  |
| Psychological distress |  |  |  |  |
| Distressed | 859 (14.6) | 331 (14.0) | 528 (15.1) | 0.27 |
| Non-distressed | 5015 (85.4) | 2036 (86.0) | 2979 (84.9) |  |

**a:** *The p-values show the significance of a Chi-square test that tests the probability of the given distributions assuming no difference of prevalence in the variable categories between non-respondents and respondents to Phase 2 survey.*

**b:** LCPA = leisure-time and commuting physical activity

**Table S3.** Rand-36 Emotional Well-being questionnaire.

|  |  | All of the time | Most of the time | A good bit of the time | Some of the time | A little of the time | None of the time |
| --- | --- | --- | --- | --- | --- | --- | --- |
| Q1^a^ | Have you been a very nervous person? |  |  |  |  |  |  |
| Q2 | Have you felt so down in the dumps that nothing could cheer you up? |  |  |  |  |  |  |
| Q3 | Have you felt calm and peaceful? |  |  |  |  |  |  |
| Q4 | Have you felt downhearted and blue? |  |  |  |  |  |  |
| Q5 | Have you been a happy person? |  |  |  |  |  |  |

a: Q = question

**Figure S1.**

**Figure S2.**

**Figure S3.**
